# Mr. Gilbert’s World Tour: Rethinking Disabled Veterans Across British Imperial Spaces

**DOI:** 10.1093/jhmas/jrad084

**Published:** 2024-03-21

**Authors:** Michael Robinson

**Affiliations:** University of Liverpool, UK

**Keywords:** Disability, Migration, Ex-Servicemen, Veterans, Shell-Shock, Prosthetic Limbs

## Abstract

This article provides a comparative analysis of the treatment of disabled First World War veterans in 1920s Britain and the simultaneous care of Imperial Pensioners residing in Australia and South Africa via the detailed administrative reports of a British civil servant, G.F. Gilbert. Imperial Pensioners were disabled veteran migrants of the British Army residing overseas. A study of these veteran populations in Australia and South Africa provides two primary insights into the broader historiography of disabled veterans. Firstly, a comparative case study helps to show the way in which cultural notions of disability were part of broader ideas of nation-building overseas. Secondly, the specific disability diagnosis category chosen as a more in-depth case study can further complicate and contradict broader assessments of national responses. This article attempts to build upon recent transnational histories of veterans by transcending national boundaries and homogenous veteran profiles with an extension in methodological scope by providing an *intra*-national case study via the Imperial Pensioner.

This article provides a comparative analysis of the treatment of disabled First World War veterans in 1920s Britain and the simultaneous care of Imperial Pensioners residing in Australia and South Africa. Imperial Pensioners were disabled veteran migrants of the British Army residing overseas. The Ministry of Pensions was the British government body in charge of distributing disability pensions and medical or rehabilitative care to disabled ex-servicemen. Australian and South African veteran departments akin to the Ministry of Pensions became agents on behalf of the British government administering care and examinations for British veteran migrants residing in their country. This article focuses on G.F. Gilbert, a Ministry employee, and his global audit of Imperial Pensioners in the mid-1920s. Gilbert was critical for inspecting Ministry policy overseas, acting as Principal Clerk in the Accounts Division of the Ministry of Pensions at London Headquarters. He was also a former Treasury employee.^1^ Pension claims and their associated administration were reduced and stable by the mid-1920s compared to the immediate post-demobilization years. Gilbert subsequently represented the Ministry in its first global audit of Imperial Pensioners and was tasked with ensuring uniformity in procedure and eradicating any administrative irregularities.^2^ While overseas, Gilbert regularly interacted with overseas pension officials and medical staff, inspected medical and rehabilitative facilities, and even personally conversed with Imperial Pensioners receiving in-patient treatment. Gilbert wrote up and dispatched his reports to his Ministry colleagues in London as his travels progressed.

Gilbert’s global tour was officially promoted as a review of the distribution of pensions and medical care overseas on behalf of the Ministry of Pensions to correct administration when necessary. Tellingly, Gilbert’s reports also contained detailed information on overseas veteran welfare systems and medical systems. Their inclusion heavily suggests that his tour was also a form of British surveillance of veteran after-care across its imperial territories. This dual purpose reflects prior research on veterans’ internationalism where cross-national points of exchange involving state actors were often multifaceted and complicated.^3^ In this article, I use the tri-national comparative case study of Gilbert’s reports on Imperial Pensioners with his simultaneous observations of veteran after-care infrastructures.^4^ Disability historians have long supported cross-national and cross-cultural frameworks to enable new perspectives on the intersections among disabled individuals, state actors, and society.^5^

While still lagging behind comparative research detailing the causes and conduct of the First World War, a burgeoning historiography exists detailing national responses to the conflict’s disabled veterans. A 2014 special virtual issue of the *Journal of the History of Medicine and Allied Sciences*, for example, marked the centenary of the outbreak of the conflict by collating influential articles. The collection’s articles focused on the war’s impact on the history of medicine and science. This virtual issue also took the opportunity to urge further research into cross-national perspectives.^6^ This article attempts to further transcend national boundaries and homogenous veteran profiles with an extension in methodological scope by providing an *intra*-national case study via the Imperial Pensioner.^7^

Case studies of individual nation-states dominate the historiography on disabled Great War veterans. Recent research has sought to build upon these works by investigating transnational exchange amongst veteran advocacy bodies. Recent research investigates the work and influence of multinational veteran advocacy groups such as the *Fédération Interalliée Des Anciens Combattants* (FIDAC) and the *Conférence Internationale des Associations des Mutilés et Anciens Combattants* (CIAMAC).^8^ Two leading historians in this field, Julia Eichenberg and John Paul Newman, note that “It is obvious that the phenomenon of the veteran’s movement goes beyond national borders.”^9^ The same transnational principle also applies to the state departments in charge of distributing veteran after-care. For example, thousands of Great War veterans exchanged via migration across the British Empire. With the funding provided by their nation of service, and following the rules and regulations of their country of origin, veteran after-care systems in a British migrant’s host nation became surrogate agents who distributed assessments, pensions, and medical or rehabilitative care.^10^ Reciprocal arrangements were established with the Ministry of Pensions also administering welfare and healthcare to foreign-born veteran migrants in Britain. As part of this imperial collaboration, numerous international visitations and exchanges like Gilbert’s occurred throughout the inter-war period. What we might call a state transnationalism of disabled veterans, however, has evaded scholarly attention.

Despite the global remit of Gilbert’s tour, this article is realistically limited to his reports on Australia and South Africa hosting two of the largest populations of Imperial Pensioners.^11^ It would also be beyond the scope of this article to discuss every individual disability in detail. For example, the Ministry’s 1925/1926 annual report listed twenty-nine primary eligible war disabilities.^12^ This article thus provides more detailed investigations into the rehabilitative infrastructure available to arguably the two most high-profile and culturally resonant disabled veteran communities, namely ex-servicemen requiring artificial leg limbs and those with a mental illness. A study of these veteran populations via Gilbert’s tour of Australia and South Africa provides two primary insights into the broader historiography of disabled veterans. Firstly, despite their apparent supposed legal equality within British welfare legislation, Imperial Pensioners were treated differently depending on the imperial space to which they migrated. This issue foregrounds the importance of individual national infrastructure in dictating the experiences of disabled Great War veterans. Secondly, focusing on two chosen disability categories complicates narratives which regularly offer broad assessments of national responses to disabled veterans. A focus on artificial leg limbs and mental health reveals how national responses to individual diagnoses could contradict a nation’s broader infrastructure. Australia offered the most regressive infrastructure regarding the distribution of artificial leg limbs despite having the most comprehensive and generous veteran welfare infrastructure. By contrast, South Africa provided the most specialised and costly infrastructure in its manufacture and distribution of artificial leg limbs. Otherwise, the nation hosted the most chaotic, disorganized and least specialized veteran welfare system out of the three nations under consideration.

Scholars have generally provided negative assessments of the British Ministry of Pensions, concluding that British Great War veterans received scant compensation and medical assistance from the inter-war British state.^13^ Cross-national comparisons have tended to solidify this narrative. Using German veteran after-care as a comparative case study, Deborah Cohen sides with the traditional view that the British state offered its veteran population paltry compensation and rehabilitation.^14^ Leading veteran historians Mark Edele, Neil J. Diamant, and Martin Crotty similarly assess that British Great War veterans looked to their Australian equivalents with “envy” during the inter-war period.^15^ Yet, this narrative can shift depending on the nation to which Britain is compared. For example, Anita Magowska utilizes the British Ministry of Pensions to argue that British pension officials were more organized and efficient compared the post-war Polish infrastructure.^16^ This article will verify how systematic comparisons can complicate existing narratives on national responses to disabled veterans.^17^

Owing to the previous works of historians researching disabled First World War veterans, a study of the Imperial Pensioner in South Africa and Australia complicates national narratives by revealing how national responses to individual diagnoses did not always match the broader national narrative. State responses to the various disabled veteran communities were often too diverse to be pigeonholed into a one-size-fits-all model regularly presented in the historiography. Research in this field often avoids a closer investigation of the nuanced experiences within veteran populations, and there remains a propensity to homogenize the national veteran experience.^18^ As medical and government officials responded differently to individual medical conditions during the First World War, particular disabilities could similarly elicit individual state responses from medico-pensions officials after the conflict.^19^

Crotty, Diamant, and Edele recently wrote that veteran communities across Britain and its Dominions “treated their veterans in broadly similar ways.”^20^ Britain, Australia and South Africa all established state-funded infrastructures to assess claims for war-related disabilities, distributed pensions, and provided state-funded medical or rehabilitative care for disabilities attributable to war service. However, as I demonstrate below, the efficiency of these pension systems varied. While the more liberal Australian system and the comparatively less generous British system ran professionally, the South African infrastructure was chaotic and disorganized in comparison. This article’s subsequent analysis of broader medical systems, including the treatment of veterans with mental illness, reflects these national narratives. Crucially, however, the inverse could also be true, as I show in the final section on artificial limbs. This intra-national case study of disabled veterans contributes to broader debates and issues within disability history by revealing what Roy Hanes recently calls its “interesting contradictions.”^21^

In addition to contributing to existing debates within veteran and disability histories, Gilbert’s observations on South Africa provide fresh insight on hitherto overlooked territories. To date, both histories of disability and veterans focus on the Global North.^22^ African veterans remain underexplored in the historiography on First World War veterans.^23^ The shortage of analysis on African nations such as South Africa appears to be due to the underdeveloped historiographies of African welfare administration, public health, and disability. George Njung, for example, assesses that the lack of written sources and perspectives of African veterans in the records can be “laborious and frustrating.”^24^ Nevertheless, this narrative is shifting with post-war studies building on scholarly works on the continent’s role and involvement in the First World War.^25^ Njung, for example, uncovers the British colonial state’s minimalization and disregard of colonial disabled Great War veterans in post-war Nigeria.^26^ Njung’s article features in a special issue of *First World War Studies* that persuasively implores First World War scholars to devote more attention to veteranhood in colonial and imperial territories.^27^ Comparing Gilbert’s report on South African infrastructure to equivalent structures in Britain and Australia provides a unique perspective on post-war disability and veteran after-care within this overlooked territory.

Focusing on institutional sources such as Gilbert’s records can be “inevitably one-sided in their account of the disabled people, presenting them as depersonalized objects of institutional action.”^28^ Activist historians of disability have often been critical of the medical model within the interdisciplinary field of disability studies. This analytical framework supposedly ensures that disabled people remain passive by centering on the medical and administrative viewpoint of the disabled mind and body which often viewed people with disabilities as requiring intervention to cure, correct, or eliminate disability.^29^ Nevertheless, echoing previous research on veterans’ internationalism, these state-centred records can also provide critical insight into how policy elites and state actors treated veterans.^30^ This article demonstrates how influential and important medical and administrative records remain especially critical to uncovering new research areas ripe for further exploration. Gilbert’s reports provide unique insight into the historical treatment of disabled migrants and how national responses and infrastructures available to disabled First World War veterans compared to one another. Gilbert’s reports can also be more nuanced than being depersonalized administrative records. Reading such sources against the grain provides instances where disabled British veterans articulate to Gilbert their lived experience of disability in their host nation.^31^ This article also integrates additional materials held in South African and Australian repositories to help mitigate an overreliance on Gilbert’s interpretation.

## PENSIONS SYSTEMS FOR BRITISH VETERANS: AT HOME AND ABROAD

By the mid-1920s, the Ministry of Pensions had an administrative headquarters in Acton, London, assisted by eleven regional offices working across the United Kingdom. The British inter-war pension system ran relatively smoothly for most of the 1920s and 1930s.^32^ The British Legion’s monthly journal, *The Legion,* featured few complaints regarding the payment of pensions.^33^ The 1919 War Pensions Act was a significant milestone in the history of the Ministry of Pensions. The legislation guaranteed disability pensions as a statutory right and introduced an independent appeal process for claimants dissatisfied with Ministry decisions. A pension of 40s 6d equated to a one hundred per cent maximum pension for a loss of two or more limbs, paraplegia, or insanity among the non-officer class with lesser disabilities met with a proportionate award. 1,199,463 disabled veterans were on the Ministry’s pension roll by 1921. The Ministry attempted to further restrict costs by introducing a seven-year time limit on claims in its War Pensions Act of 1921, debarring any fresh claims after 1 September 1928. The Ministry also distributed Final Awards via lump sums or definitive pension rates aimed at veterans with more minor disabilities and which had supposedly reached a permanent state. Broader government retrenchment initiatives in public spending were labelled the “Geddes’ axe,” enforcing a host of tax increases and economic reductions across the public sector.^34^ This wide-scale retrenchment dampened accusations of state neglect of the British disabled veteran. British leading veteran organizations also merged in 1921 to form the British Legion. The veteran charity subsequently struggled to combat the retrenching measures included within the Ministry’s 1921 Act.^35^ Gilbert’s reports on Australia and South Africa demonstrate the importance of the lobbying power and persistence of veteran advocacy bodies in shaping state policy.

Many British migrants travelled overseas via the Overseas Settlement Committee and its associated free passage schemes between 1919 and 1922. This scheme provided men and their families with state-funded third-class boat passages to the dominion or colony of their choice. An unknown number of migrants also self-funded their migration. The medical screening of veterans remains unclear. Only Kent Fedorowich has assessed the experiences of British ex-servicemen who migrated to the “White Dominions” of Australia, New Zealand, Canada, and South Africa.^36^ Fedorowich’s study focuses on veterans not disabled by war service ensuring that we still know very little about the fate of disabled ex-service migrants. Migratory restrictions in Australia and South Africa emphasized race over medical suitability with broad categorizations open to individual interpretation by migratory officials.^37^ It remains difficult to uncover what precise medical checks were processed regarding the health of individual British veterans wishing to migrate. Regardless, the Ministry of Pensions’ policy continued to cater to disabled British Great War veterans overseas. At its headquarters in London, the Ministry had a special branch for “overseas” cases.^38^ 23,000 Imperial Pensioners resided across more than one hundred countries by 1936, equating to less than two percent of the disabled veteran community living across the UK. Post offices regularly distributed pension payments to British veteran migrants verifying its importance in transferring funds amongst imperial subjects and overseas communities.^39^ Gilbert’s global tour was approved and organized by the Ministry of Pensions to provide the first global inspection and auditing of this transnational system.

Gilbert arrived in Australia on 7 July 1926.^40^ The Australian system of pensions distribution broadly reflected the Ministry of Pensions’ infrastructure. The Australian federal government established the Repatriation Commission in 1920 bound by statutes and regulations, regional officials, and medical officers working on its behalf across the country.^41^ The Repatriation Commission also administered medical and rehabilitative care to disabled veterans via specialized and exclusive departmental hospitals run by state officials rather than the federal government. Repatriation Commission facilities catered for 70,128 disabled veterans of the Australian Imperial Force (AIF) alongside 3,236 Imperial Pensioners by 30 June 1926.^42^

Crotty, Edele, and Diamant describe Australian veterans’ inter-war homecoming as “truly exceptional” and the “best case scenario” in the history of veteran community homecomings.^43^ The wider historiography generally endorses this view.^44^ Verifying the Australian system’s generosity, Gilbert noted that medico-pensions officials regularly attributed claims for transient and progressive conditions as pensionable disabilities attributable to war service which British officials would have denied. For example, Gilbert reserved caution regarding the Australian system’s assessment of claims for veterans with tuberculosis processed on behalf of British and Australian claimants. He adjudged the three-men Repatriation boards working in each of the six states to be far too liberal in accepting that inconclusive claims for this disability were due to war service. Gilbert observed the acceptance of dubious claims merely with the proviso that the claimant served near the front line.^45^

Gilbert referenced other liberal legislative differences in Repatriation Commission policy including the lack of any definitive time limit policy or system of distributing final awards to remove veterans from the pensions roll. His report cited public opinion and the socio-political context to explain Australian policy’s benevolence: “This liberality was born of the spontaneous sympathy and generous impulses of the Australian people, but I am afraid that it is now kept alive and fostered by the exigencies of politics. The political element apparently enters far more into pension matters in Australia than in Great Britain.”^46^ Historians attribute the political element referenced to the “Anzac Legend.”^47^ Established in 1901, the Commonwealth of Australia’s participation in the First World War helped to give birth to Australian nationhood subsequently placing its Great War veterans in a sacred and elevated socio-political position.^48^ Historical research similarly identifies the Returned Sailors and Soldiers’ Imperial League of Australia (RSSILA) as an “effective champion” for the disabled Australian veteran in the historiography. The RSSILA were able to effectively capitalize on the political currency attached to military service to extract more favorable policy concessions from Australian authorities.^49^ Gilbert also held that it was indisputable that veteran groups such as the RSSILA could subsequently exert far more political pressure on governments to provide policy concessions to Australian veterans compared to Britain. For example, returning to his earlier skepticism of dubious tubercular claims being adjudged as being attributable to war service, Gilbert held that veteran bodies had extracted a liberal policy of interpretation from the incumbent coalition government in the build-up to the 1925 federal election.^50^

Gilbert’s observation of the political constitution of Australia provides another potentially critical but hitherto overlooked feature helping to explain the country’s comparative benevolence. Unlike Britain’s sole House of Parliament elected by popular franchise, Gilbert theorized that Australian branches of federal and state governance enabled the cultural currency attached to veterans to become a key electoral issue at both the national and local levels. Veteran bodies effectively capitalized upon this increased democratic opportunity before elections. Several high-ranking Australian officials agreed with Gilbert’s viewpoint, including the chairman of the Repatriation Commission, J.M. Shemmens.^51^

With the Repatriation Commission acting as an agent on behalf of the Ministry, Repatriation officials or associated Medical Officers processed pension assessments to decipher a British migrant claimant’s eligibility and grading for a pension and medical treatment. As was the case in Britain, renewal awards occurred six months before the expiration of an Imperial Pensioner’s award in Australia. Over 2,000 assessments occurred in the year before Gilbert’s arrival. While noting administrative irregularities, Gilbert informed colleagues in London that he was “favourably impressed” with the Ministry’s arrangements in Australia and the state of pensions administration in the country.^52^ The Repatriation Commission took pride in Gilbert’s praise for their professional and organized veterans’ pensions system being able to amalgamate British veteran migrants relatively seamlessly.^53^ Indeed, their annual report for 1923 noted the “very considerable” work undertaken to establish and arrange medical examinations and distributed pensions for the British disabled veteran resident across Australia.^54^

Like Australia, South African veteran authorities distributed payments to Imperial Pensioners as surrogates on behalf of the Ministry. The office of the Commissioner for Returned Soldiers and the Military Pensions Board mutually distributed veteran after-care. The Commissioner for Returned Soldiers seemingly catered to the entire ex-service community. The Military Pensions Board, established in 1917, appears to have dealt exclusively with the payment and medical care of South African disabled veterans and Imperial Pensioners. With a centralized headquarters located in Pretoria, the Military Pensions Board performed all executive functions under the remit of the South African Treasury. This setup differed from the separate and distinct state department for disabled veterans operating in the form of the Ministry of Pensions in Britain and the Repatriation Commission in Australia. The Military Pensions Board’s boarding and assessment of South African veterans and Imperial Pensioners were undertaken in regional pension offices located across the country.^55^

On Gilbert’s arrival to Pretoria on 16 November 1926, 8,824 disabled Great War veterans of the South African Army alongside 1,759 Imperial Pensioners were under the remit of the Military Pensions Board.^56^ Gilbert wrote that South African pension legislation and rehabilitation services were “largely founded on our own.” South African medical and civil servants had visited London in 1918 for dialogue with Ministry of Pensions’ officials while designing their domestic infrastructure.^57^ South Africa’s rehabilitative policy subsequently aligned more with British policy being less liberal than the Australian provisions.^58^ Gilbert spent eight months in South Africa. This constituted the most prolonged time spent in any nation during its global tour, doubling the time previously spent in Australia despite South Africa hosting less than half the number of Imperial Pensioners. Gilbert did not anticipate such a lengthy residency. The disorganization and inaccuracy of procedure evident in the dominion after his arrival compelled a more in-depth intervention.^59^ Four years earlier, information produced for the International Labor Office similarly lamented the imprecise data and lack of available information on disabled veterans living in South Africa.^60^ By Gilbert’s arrival, South African officials were unaware of the need to differentiate Imperial Pensioners from their veteran community and administer separate British and South African policies. The inability to locate pensions officials knowledgeable of domestic policy and practice consistently dismayed Gilbert. The unfamiliarity regarding Ministry guidelines for treating Imperial Pensioners led Gilbert to tell his Ministry colleagues in London that there was “considerable ignorance of our instructions.”^61^ There was apparently little concern about governing the payment of pensions and allowances to Imperial Pensioners with very few officials having even read Ministry instructions regarding Imperial Pensioners.^62^

This laxity regarding disability assessments was financially beneficial to the British veteran but came at a cost to the Ministry, with Gilbert concluding, “It was abundantly evident to me that Imperial Pensioners had not suffered. On the contrary, I had ample evidence that they received every courtesy and consideration … [this] has certainly been to the advantage of the Pensioner but hardly of that to the British taxpayer.”^63^ A representative of the British Empire Services League (BESL), a veterans charity representing both Imperial Pensioners and South African veterans, relayed their satisfaction to Gilbert regarding the country’s distribution of pensions.^64^ Evidencing the lack of understanding of British policy, which benefited migrants but came at a financial cost to the British state, South African officials were unaware that the Ministry had even imposed a seven-year time limit on claims.^65^ This administrative oversight of a central pillar of British policy facilitated the medical care of ineligible British ex-servicemen at Ministry expense. This costly lapse included, for example, funding one ineligible British veteran to travel 1,200 miles to be assessed by a medical specialist.^66^

Like the description of Australian conditions, the South African report reinforced the contingency of domestic arrangements in dictating administrative policy towards Imperial Pensioners. For example, the records of the Military Pensions Board’s central office in Pretoria complained in 1926 that medical assessment staff were haphazardly granting authority to compensate and care for veterans for medical conditions unrelated to war service. The South African officials urged greater discretion and control over the administration of state-funded treatment to distribute pensions and treatment and carry out disability assessments.^67^ Gilbert similarly noted an absence of any consistent or settled criteria regarding pension eligibility. Reflecting the austere mind-set of his employers, Gilbert articulated persistent irritations as to the unnecessary costs distributed in South Africa on behalf of the Imperial Pensioner.^68^

Gilbert’s report again verified the critical importance of broader public opinion and the socio-political context in dictating veteran policy. Gilbert concluded that South Africa’s approach differed from Britain by prioritizing the veteran claimant over the taxpayer. Like Australia, the political pressure applied by veteran bodies extracted favorable concessions from the South African state.^69^ An earlier Ministry observational report on South Africa written four years earlier repeated this feature. With South Africa’s Military Pensions Board acting as a sub-department subservient to the Treasury, it did not employ its own full-time medico-pensions officials to carry out assessments as was often the case in Britain and Australia. Instead, doctors working for the South African Public Health Department assessed the attributability of a veteran’s disability claim on behalf of the Military Pensions Board. Gilbert’s report argued that this out-sourcing influenced examiners to be overly liberal when conducting assessments to appease personal and public sentiment rather than objectively judge claims on behalf of the state. In addition to regularly favoring the claimant over the state, independent Public Health Department doctors could not then have their verdict challenged by either the Military Pensions Board or the Treasury.^70^

Ministry officialdom long castigated South African policy. Gilbert quoted the previous Ministry observational report questioning whether South African officials even followed any policy at all. The earlier report offered an imperial explanation to explain the ad-hoc conditions in South Africa. The report blamed the impulsive and short-term thinking inherent amongst South Africans who were liable to “live from hand to mouth” and to “scorn the wisdom of their ancestors, and to take shortcuts.”^71^ Studies on the Ministry of Pensions’ observation of disabled veterans in India and Ireland similarly describe its use of derogatory imperial discourse regarding veteran behaviour to explain failures or shortcomings in state policy.^72^ Gilbert’s report provides a more practical explanation. First, all Great War veteran policy extended itself to another veteran community, namely Afrikaner veterans of the Anglo-Boer conflicts. This extension of welfare rights aimed to placate Boer nationalist sentiment. Both Ministry reports from 1922 and 1926 recognized that catering for separate veteran communities distinguished by race, language, politics, and conflict reduced burdens of proof amongst claimants and affected overall administrative efficiency. In addition, South Africa had the peculiarity of having an administrative capital in Pretoria and a parliamentary capital in Cape Town to satisfy both Boer and British loyalist sentiment respectively. This compromise, in the Ministry’s two observational reports’ opinion, further impeded efficient policymaking and the administration of veteran welfare programs.^73^ Like Australia, the political constitution of South Africa may help explain its differences from Britain in veteran policymaking and welfare administration.^74^

The extraction of favorable concessions from state departments was not a universal experience across Africa. White First World War and Boer War veterans in South Africa seemed better able to pressure their government to improve disability welfare policy and infrastructure in a way that colonial veterans in post-war British Nigeria, for example, were not.^75^ This differentiation offers an example of how further research on veterans returning to Africa can provide insight into the critical but nuanced intersections of race, citizenship, and army service and their influence on welfare state policy and veteran after-care.^76^ Regardless, Gilbert reported that an organized and efficient administrative setup was not consistent among combatant allied nations. While Britain and Australia were ostensibly able to establish relatively organized administrative systems, carry out medical assessments, and distribute pensions, South African infrastructure was described as providing a more disorganized setup for Imperial Pensioners to share alongside South African veterans, albeit this disorganization could also be financially beneficial to veterans in South Africa. Broader national medical infrastructures reflected these pensions systems.

## MEDICAL INFRASTRUCTURES

Ministry medical and rehabilitative infrastructure operating throughout the UK treated almost 150,000 pensioners by March 1921 across 113 hospitals. Specialist in-patient care in Ministry hospitals and out-patient treatment in Ministry clinics provided this treatment for a wide range of war-related conditions.^77^ There is little research to date on the Ministry’s medical system, albeit *The Legion’s* back catalogue provides few complaints regarding the standard of care on offer to eligible veterans. To contribute to the cost of in-patient care, an in-patient was deducted 19s from their state-funded pension. This welfare came in the form of a “Treatment Allowance” which automatically (but temporarily) increased the levels of an in-patient veteran’s pension to a maximum rate. This uplift encouraged employed veterans to take leave from work to receive institutional care.^78^ The retrenching War Pensions Act of 1921 and its cost-cutting drive included closing down or downsizing Ministry facilities. In its place came greater use of municipal hospitals and National Health Insurance-funded General Practitioners (GPs) who provided primary medical care to pensioners, paid for by the Ministry.^79^ Just nineteen Ministry hospitals were operating by March 1927 with admission reserved for acute and urgent cases.^80^

Most disabled Great War veterans in Britain appear to have joined the majority of other male workers covered by the National Health Insurance Act (1911) in receiving primary care through their GP by the time of Gilbert’s world tour. Indeed, his report on Australia compared how British policy relied more on outsourcing medical care to the National Health Insurance system to provide the bulk of medical care to veterans. By contrast, the Repatriation Commission maintained closer contact with its veterans and directly provided the majority of medical care with veterans located in and around the more populated areas of Australia. He and another Ministry colleague, W.F. Smith touring Australia a decade later, theorized that the increased liability for Repatriation Commission officials to recommend Australian and Imperial Pensioners for treatment was due to country’s lack of health insurance coverage which was otherwise available to veterans in Britain.^81^ Reflecting the assessment of pension claims in South Africa, Gilbert was skeptical regarding the provision of medical care for South African veterans with inconclusive claimants being recommended for state-funded treatment.^82^ As veterans in Australia and South Africa were more likely to be admitted for state-funded medical care, this article now turns its attention to Gilbert’s descriptions of the contrasting standards of care then on offer.

Exclusive Repatriation General Hospitals with attached out-patient clinics existed in five out of six states.^83^ State lunatic asylums hosted veterans in either segregated blocks or segregated buildings. Local hospitals or Repatriation funded local medical officers treated disabled pensioners in more isolated areas. 4,421 Australian ex-servicemen received Repatriation Commission-administered in-patient care with 1,560 undergoing outpatient treatment by June 1926.^84^ Imperial Pensioners shared state-funded facilities with Australian veterans. 651 Imperial Pensioners were receiving treatment on Gilbert’s arrival. Most Imperial Pensioners received treatment in Australia for diseases and illnesses such as bronchitis, tuberculosis, heart problems and psychoneurotic ailments. Imperial Pensioners appear to have mostly attended a Repatriation Commission out-patient clinic twice a month to collect alleviating medicines.^85^ Regarding in-patient treatment, the bulk of provisions constituted 130 Imperial Pensioners receiving medical care for tuberculosis with ninety-eight apiece for bronchitis/asthma and neurasthenia.^86^

Gilbert praised the Repatriation Commission’s in-patient facilities and their regularly attached outpatient clinics. He complimented Repatriation facilities on being ideally located and accessible in the nearby suburbs of capital cities with ample medical equipment. His report notes the uplifting appearance of veteran facilities and the considerate care on offer with available bowling greens, croquet lawns, and billiard tables for recreation with up-to-date canteens offering good portions of a varied diet. Gilbert spoke with several British in-patients during his tour of Repatriation facilities who relayed their satisfaction with their care reinforcing Gilbert’s positive assessment. However, the aforementioned British policy to deduce 19s per week from Imperial Pensioners to contribute to their maintenance cost was a source of grievance amongst British migrants. This complaint intensified due to the small number of British veterans sharing facilities with Australian veterans for whom no such deduction occurred.^87^ These complaints again speak to the miserliness of British policy when compared to Australia.

In South Africa, Gilbert considered the medical infrastructure available to Imperial Pensioners to be inferior. The Military Pensions Board’s subservient and non-exclusive role within the South African government also ensured that no exclusive facilities existed for the treatment of disabled veterans. Instead, ex-servicemen were accommodated within existing public or military medical facilities alongside non-veterans. South Africa’s Public Health Department wrote that 2,100 military cases were treated in 1926, amounting to a daily average of 255 men during the previous year.^88^ The country’s disorganized administration proved unable to provide Gilbert with figures informing him of the specific numbers of disabled South African and British veterans receiving medical care. Information on South African policy and infrastructure administering medical care and rehabilitative facilities was severely lacking. Gilbert even questioned whether there was any national policy on veteran after-care due to the dire state of disorganization and dearth of available information.^89^ He was similarly unimpressed with the available military medical institutions shared by South African veterans, Imperial Pensioners, and service personnel of the South African Armed Forces. Overall, Gilbert described South African facilities as underfunded, overcrowded, and unsuitable in-patient facilities.^90^

The reduced number of veterans requiring care explains the lack of exclusive veteran hospitals. Just fifty pensioners received care in the Roberts Height, Pretoria, and Wynberg, Cape Town, military hospitals. This was a sizeable reduction from the 180 under treatment five years earlier. These hospitals included chronic, unemployable, and even some homeless Imperial Pensioners and South African veterans. Some remained under care despite it being clear to Gilbert that continued treatment provided no benefit.^91^ In the absence of a workhouse system that could host ageing and destitute veterans in Britain, it appeared that Roberts Height and Wynberg military hospitals in South Africa took up this de-facto role. In comparison to Britain and Australia which increasingly prescribed out-patient treatment as the inter-war year period progressed, medical and pension officials rarely prescribed out-patient care due to the lack of appropriate facilities and infrastructure.^92^

Without segregated medical facilities exclusively available for veterans, Imperial Pensioners shared available conditions with South African veterans and non-veterans alike. Gilbert’s report on South Africa noted how providing additional and improved hospital facilities for South African society had long been “a burning question.” He observed that overcrowding and insufficient medical staff and appropriate equipment were persistent and widespread national problems. Gilbert’s residency coincided with the writing up of an appointed government commission on how to address these long-standing and vital issues. Gilbert’s report verified the need for increased state intervention. For example, on visiting Roberts Heights Military Hospital, he described how the facility had a bad reputation for lacking discipline and being uncomfortably hot during the summer due to its wooden and iron framework. The hospital had no x-ray machines or laboratory facilities with a shortage of recreational facilities to help stimulate patients. Gilbert discussed the facilities with the four Imperial Pensioners residents during his visit to the hospital. British in-patients relayed little enthusiasm for their care. They complained bitterly about the hospital’s disorganization, emphasising the substandard dietary provisions and the absence of any comforts or adequate treatment.^93^ Due to the aforementioned ignorance of British policy, Union officials had also incorrectly implemented Ministry policy by providing British migrants receiving in-patient care a one hundred per cent pension when their medical admission and subsequent treatment was not war-related.^94^ This additional financing again speaks to British migrants in South Africa benefiting financially from the lack of understanding of correct procedure in the dominion. Gilbert sought to intervene and correct this administrative oversight to benefit the Ministry but showed little similar inclination to help improve the lot of Imperial Pensioners receiving care in sub-standard facilities.^95^

Australia and South Africa’s broader medical infrastructure mirrored their administrative pensions infrastructure, presenting a broad narrative of organization and liberality in the former and chaos and detachment in the latter. A study of war-related mental disabilities supports these national narratives. “Shell-shock” had become the catch-all medical term in Britain to categorise mental symptoms during the First World War. The term then fell out of favor as a diagnostic category as it became clear that servicemen could display psychological injuries without experiencing shell-fire or even front-line service. The pre-war diagnosis of neurasthenia returned to the medical nomenclature becoming the primary diagnosis for Ministry medico-pensions officials to denote any psychoneurotic condition in the post-war period. An estimated 65,000 British veterans were receiving a pension for neurasthenia in the UK by 1921.^96^ The Ministry initially established twenty exclusive in-patient facilities and forty-eight out-patient clinics able to accommodate neurasthenic pensioners. There were 2,951 in-patient beds to treat neurasthenia and facilities to accommodate 6,975 neurasthenic pensioners in out-patient facilities in 1921. The retrenching 1921 War Pensions Act again reduced this infrastructure. Just 1,373 in-patient beds with space for 541 out-patients were in place by 1927. There was a further reduction of abreaction therapy pursued in remaining facilities with a keener focus on a policy of “hardening.”^97^ Hardening provided more vocational training in place of medical treatment to help transition the shell-shocked veteran to employment post-discharge.^98^ Despite tentative steps to provide progressive psychotherapeutic treatment during the immediate post-war years, neurasthenic veterans were thus mostly provided with pensions but received little medical intervention after 1921.^99^

Institutionalized Great War veterans were an additional administrative and medical category under the charge of the Ministry of Pensions. Lunacy was graded at a maximum one hundred percent pension akin to losing two or more limbs. The Ministry established the Service Patient scheme to counter the stigma attached to lunacy and institutionalization.^100^ Categorized akin to private patients, Service Patients wore distinct civilian clothing to differentiate them from the regular asylum population to help spare them from the stigma of pauper lunacy. They also benefited from a weekly allowance of 2/6d for “additional comforts.”^101^ With an estimated ten to twelve Ministry institutions quoted as necessary to cater for “insane” veterans across the UK, the Ministry ruled out their establishment from the outset assessing that the associated costs would be too expensive.^102^ There were an estimated 9,481 Service Patients in UK institutions in 1926.^103^ Previous research into the Service Patient scheme in inter-war England, Scotland, and Ireland diminishes its benefits. Instead, the subjective conditions within individual asylums, shared by veterans and non-veterans alike, appeared far more consequential than their legal status.^104^

Australia provided facilities for neurasthenic veterans within its five Repatriation General Hospitals. These facilities offered a varied range of treatments including psychoanalysis, counselling, hypnosis, convalescence, and physiotherapy.^105^ Repatriation Commission annual reports during the immediate post-war years attest to the potential for positive results via this prolonged individual psychotherapeutic treatment.^106^ As was the case in Britain, Australian officials became increasingly skeptical about the curability of neurasthenic pensioners believing that the best results had already been achieved by the mid-1920s.^107^ Only one Repatriation General Hospital had a separate ward exclusively reserved for neurasthenics by Gilbert’s arrival. His subsequent report informed Ministry officials that the treatment of neurasthenics in Australia reflected the same problems evident in Britain. Gilbert relayed similar frequent lapses in the recovery of psychoneurotic pensioners repeating suspicions in Britain that Australian veterans also prolonged their hospitalization for its material and financial benefits. Following his interaction with Australian officials, Gilbert wrote how the Repatriation Commission was similarly “fed up” with the lack of progress made with neurasthenic pensioners despite costly in-patient and out-patient care remaining on offer in the country.^108^

In contrast to Britain, exclusive segregated blocks or wards existed within larger public mental health facilities to separate and distinguish insane ex-servicemen from the wider in-patient population. This separation fitted the Repatriation Commission’s broader ethos of preferential treatment reserved for Australian ex-servicemen. Compared to non-veteran institutionalized populations, former AIF veterans received a more expensive standard of care from carefully selected and higher-paid staff. The facilities also provided a better diet, more recreational events and day trips, and veterans were not required to perform any labor duties. This segregation had symbolic and practical benefits for veterans with their distinction helping to spare them and their families from the stigma of hereditary and degenerative mental illness.^109^ Gilbert also held that Repatriation facilities for ex-servicemen were superior to public asylums available in Australia and Britain. The sixty-three insane Imperial Pensioners residing in Australia subsequently received better treatment in brighter and more cheerful institutions afforded to their similarly-afflicted ex-comrades in Britain. While in Melbourne, Gilbert visited the segregated facility for mentally-ill veterans on the grounds of Royal Park Hospital for the Insane hosting three Imperial Pensioners. Gilbert complimented the “lavish provision made for the comforts of the patients, the appointments being really suggestive of a first class hotel.”^110^

Due to the absence of statistics available on South Africa, it is unclear how many of the 8,824 South African veterans received a disability pension for a mental illness. The scant research into mentally ill First World War veterans in South Africa indicates an absence of specialist infrastructure or knowledge to assist mentally ill veterans. Public mental hospitals received mentally disabled veterans owing to the lack of expertise and suitable infrastructure.^111^ In many aspects, the South African experience of post-war combat neurosis was unexceptional. South African medico-pensions officials relayed the same scepticism evident in Britain and Australia as to the curability of neurasthenic veterans. Located in South Africa, Gilbert wrote to his London-based colleagues informing them that the “neurasthenic presents the same difficulty and gives the same trouble in the Union as he does at home. He rarely responds to treatment … [the] number of permanent cures is practically negligible.”^112^ South African officials shared Gilbert’s skepticism. In correspondence sent to all advisory boards and committees as early as February 1919, the Commissioner for Returned Soldiers wrote of the immense difficulty in treating shell-shocked veterans due to the subjectivity of symptoms apparent amongst individual claimants.^113^

Repeating a phrase often utilized by British medico-pensions officials, and echoing observations of Australian practice, South African officials articulated accusations of “pensionitis” amongst its neurasthenic veterans. Pensionitis referred to neurasthenics whose condition was supposedly constitutional in nature. Such men avoided recovery or improvement in order to continue receiving a disability pension. One Military Pensions Board Medical Officer told Gilbert of the prevalence of pensionitis in the country stating that, in his medical opinion, the neurasthenic veteran was “absolutely rotten.”^114^ South African officials relayed how they were also “heartily sick of the neurasthenic and would be heartily glad to be rid of him.”^115^ By 1926, South African authorities provided many psychoneurotic veterans with a lump sum gratuity and severed all administrative and legal ties with them rather than remaining eligible for their prolonged maintenance and upkeep.^116^ Gilbert told his Ministry colleagues in London of the difficulties of effectively and fairly compensating and rehabilitating neurasthenic pensioners in South Africa. He cited this difficulty to verify how Britain’s continuous surveillance and distribution of pensions were preferential in the treatment of neurasthenic veterans.^117^

Unlike Britain and Australia, there was no opportunity for neurasthenic veterans in South Africa to receive specialized care in exclusive facilities. Military hospitals such as Roberts Height military hospital or public institutions often received neurasthenic veterans requiring in-patient treatment. These facilities provided negligible specialist psychotherapy or even segregated the mentally ill veteran from physically ill civilian and soldier patients. Addington Hospital, Durban, was one public hospital hosting neuropsychiatric veterans, albeit with separate temporary accommodation for ex-service personnel. The hospital was lacking in comfort with its veterans mixing openly with the much larger civilian patient profile including elderly and chronic civilian patients.^118^

Regarding insane veterans in South Africa, nursing homes or underfunded and unfurnished public mental hospitals accepted numerous veterans with minimal specialist care, comfort, recreation, or dietary innovation with little expectation of recovery. Unlike the Service Patient scheme in Britain, and the segregated facilities in Australia, which were again praised as “special institutions” in Gilbert’s report on South Africa, veterans in South Africa received no additional privileges or recognition for their mental disability.^119^ Describing South African facilities hosting insane veterans, Gilbert wrote: “apart from workhouses at home, I have seldom been in a more uninviting institution.”^120^ Gilbert’s derogatory observation in South Africa reflects tragically on the ex-servicemen in Britain who *did* enter an inter-war British workhouse.^121^ Regardless, previous research into institutionalized First World War veterans stresses the increased likelihood of stimulating and humane in-patient facilities positively affecting in-patients’ bodily and mental state.^122^ Supporting this feature, the report wrote that the desolate facilities in South Africa, providing meagre meals and a lack of home comforts, ensured that its in-patient population appeared poorly dressed, weak and depressed.^123^

A cross-national comparison of the treatment of insane veterans demonstrates how the country chosen as a comparative case study is crucial to dictating judgments on national policy. The British Service Patient scheme seems miserly and insignificant in comparison to the Repatriation Commission’s segregated facilities. Great War veterans in sub-standard British asylums would surely have benefited from having segregated or exclusive institutions had the Ministry not initially ruled out their establishment due to their associated costs. Further demonstrating the Ministry’s disregard of insane veterans, Gilbert dismissed Service Patients as “second proviso” pensioners in his proceeding report on New Zealand.^124^ However, Service Patients in British asylums experienced superior care and recognition than what was otherwise on offer in South Africa. Integrating South Africa, rather than Australia, provides an inverse interpretation of the British Service Patient scheme enabling a more generous assessment of a policy hitherto heavily maligned in the historiography.

Regarding neurasthenia, Britain, Australia, and South Africa had a closely aligned outlook with each nation’s medico-pensions hierarchy remaining skeptical of the neurasthenic veteran’s curability by the mid-1920s. Yet, each nation had contrasting policies and infrastructures catering for psychoneurotic veterans. These variances are important. Despite cynicism about the benefits of psychotherapy and abreaction treatment, Gilbert claimed that its continuance in Australia had provided “some rather remarkable cures” albeit to a minority of veterans.^125^ Verifying its benefits, the Repatriation Commission’s annual report for 1925 includes a lengthy verbatim report by a medical official attesting to the benefits of continued abreaction therapy.^126^ This positive assessment echoes the benefits of psychotherapy undertaken in Britain during the immediate post-war years.^127^ This issue speaks to an essential matter; the success of medical care regarding neurasthenic pensioners should not exclusively be judged by the attainment of complete cure and recovery. The continuation of Repatriation facilities for psychoneurotic veterans in the 1920s may, at the very least, have helped to stabilize veterans’ condition or aid them in better coping with their psychoneurotic symptoms and by preventing them from deteriorating further.^128^ Surviving archival records of the Ministry of Pensions, the Repatriation Commission, and the Military Pensions Board continuously overlook the importance of this more modest aspiration. By 1926, Australian and Imperial Pensioners in Australia had an increased chance of this more progressive form of care than in Britain and, most certainly, than in South Africa.

The treatment of mentally ill veterans in Britain, Australia, and South Africa aligns with broader national administrations of pensions and medical infrastructures. Historians should, nevertheless, avoid painting national responses to disabled veterans with a broad brush. This article’s final section on artificial limbs illustrates this vital caveat. An investigation of artificial limbs provides a contrasting instance of British infrastructure and policy bettering what was otherwise offered in Australia. Further advancing the importance of assessing disabilities individually, South African infrastructure was then superior to both Britain *and* Australia despite the aforementioned chaotic pensions administration and substandard medical structure otherwise available. A closer analysis of artificial limbs illustrates the “interesting contradictions” often inherent within disability history.^129^

The standardization of the manufacturing process and the emergence of metal in place of wood led to the rapid increase in artificial limb quality in post-war Britain. The technological advancement of metal limbs, developed by private companies such as Dessouter Bros Ltd, ensured that several firms across the UK provided artificial limbs to the Ministry by 1925. The Ministry of Pensions, the British Legion, and the *British Medical Services of the Great War Official History* attested to the versatile metal limb’s durability and flexibility.^130^ In 1936, reflecting on its previous two decades, the Ministry of Pensions described its distribution of artificial metal limbs as “one of its outstanding successes.”^131^ Historical research into Ministry limbs verifies the rapid advancement in artificial metal leg limbs after the First World War.^132^ The Ministry’s artificial limbs became innovative enough to be distributed to limbless civilians and children alongside the veteran population.^133^

Twelve Imperial Pensioners were provided with Australian-made artificial limbs in the year before Gilbert’s inspection although the overall number of limbless Imperial Pensioners residing in Australia is unclear. Visiting five artificial limb manufacturing sites across Australia, Gilbert held that the wooden artificial limbs possessed by Imperial Pensioners and Australian veterans were inferior to the light metal limb widely distributed in Britain.^134^ Prior research by Joanna Bourke confirms this assessment finding that limbless Australian veterans struggled in their rehabilitation during the inter-war years. The Repatriation Commission distributed wooden limbs produced by its own factories established in each state. Unlike Britain, which took steps to specialise in developing and manufacturing artificial limbs during the First World War, the Australian response lacked the equivalent expertise and wartime focus on orthopaedic care. As a result of this standing start, Australian limb-making factories continued to distribute wooden limbs, at the expense of lighter and more flexible lighter metal limbs, by Gilbert’s arrival. Limbless veteran associations in Australia subsequently complained to state authorities about veterans having to toil in outdoor work in hot weather with heavy and cumbersome wooden legs.^135^

The Repatriation Commission observed artificial limb manufacturing and distribution in other combatant nations during the mid-1920s, noting that metallic limbs regularly distributed overseas were preferable to their wooden devices. They subsequently wrote of their ambition to further develop, produce, and administer its own metal limbs in the coming years.^136^ Bourke asked in 2008: “to what extent were [limbless] Australian soldiers aware of the differences between their treatment and that of their British counterparts?”^137^ More research on this unanswered question is now needed. Regardless, this article’s initial analysis complicates previous assessments that have portrayed the Repatriation Commission as a benevolent institution. This thesis builds upon recent research on the Repatriation Commission’s treatment of ageing Australian former military nurses after the First World War which has also begun questioning this established narrative.^138^

The manufacture and distribution of artificial limbs appears superior in South Africa despite the otherwise disorganized pensions administration and medical infrastructure. Gilbert visited the South African government’s artificial limb factory in Johannesburg which imported the metal duralumin parts from Britain to construct individual models providing another example of the Ministry of Pensions’ influence on South African policy and infrastructure. Gilbert’s report described South African artificial limbs as being “well-made and excellently finished.”^139^ Limbless veterans attended the Johannesburg factory for personal customised fittings. The two-week residency of limbless Imperial Pensioners for fitting cost the Ministry £65 per veteran. Gilbert praised customised surgical boots as “works of art.”^140^ Several limbless Imperial Pensioners in attendance at the factory also relayed their satisfaction with their artificial limbs and the rehabilitative care they had received.^141^

The Military Pensions Board’s Director of Medical Services in South Africa told Gilbert that he regarded his nation’s appliances as vastly superior to British models, especially their surgical boots. The South African official articulated how he was “rather indignant” at the inferior British model boots that British migrants arrived with who he believed had been “fobbed off” by the Ministry and were subsequently unable to walk freely with their British-made appliances.^142^ He subsequently thought it would be a “gross breach of faith” to reduce the standard of their artificial limbs to those provided in Britain and Australia.^143^ The South African Public Health Department also took pride in their artificial limb service. It held that their models surpassed any other combatant nation’s quality and limbless veterans were satisfied with their appliances.^144^

South African medico-pensions officials advocated for their sturdier surgical boots as many South African ex-servicemen engaged in intensive outdoor agricultural labor, justifying pursuing costlier models.^145^ Gilbert noted that the associated costs with artificial limb replacements in South Africa could double or even treble the equivalent costs in Britain. The Ministry disregarded any potential to replicate South African policy, despite its benefits to limbless veterans further revealing the Ministry’s austere mind-set, on account of its associated costs. Gilbert went even further to restrict Ministry costs and emphasized to South African officials to enforce procedure with British policy when fitting artificial limbs. Gilbert’s austerity on behalf of his employers extended to instructing South African officials to cease personalized fittings for limbless Imperial Pensioners at the artificial limb factory in Johannesburg despite its clear benefits to veterans.^146^

An analysis of artificial limbs across Britain, Australia and South Africa illustrates how nations do not always fit seamlessly into linear and generalized narratives. The variable treatments for limbless Great War ex-servicemen sharply deviated from broader administrative and medical infrastructures. The historiography regularly depicts Australian Great War veterans as a best case scenario in the homecoming treatment of veterans.^147^ By comparison, British First World War veterans are described as looking on enviously toward their Australian counterparts.^148^ While Gilbert’s overall report endorses this broader narrative, an analysis of artificial limbs complicates this account. While hosting an otherwise chaotic administration of pensions and an inadequate medical infrastructure, the standard of artificial limbs in South Africa then bettered anything on offer in Britain despite the latter’s specialization and pride in its artificial limb capacity. Owing to the prior research on disabled First World War veterans, historians can now properly investigate individual disability diagnostic categories to facilitate a fuller understanding of the nuances involved in national responses to disabled Great War ex-servicemen.

## CONCLUSION

This article’s triangulation of three allied nations, spanning three continents and incorporating broader pensions administration and medical infrastructures, supports historians who argue that ex-service populations were not one homogenous or monolithic block.^149^ The same linear path is similarly problematic for all disabled populations in past societies.^150^ Like more comprehensive analyses of welfare state development, the historiography on First World War veterans often relies on isolated national case studies at the expense of transnational histories analysing the international transferal of ideas.^151^ Prominent areas worthy of further exploration include assessing overseas territories’ viewpoints and the impact of British visitors on overseas domestic policy. Gilbert’s visit enabled overseas medico-pensions officials to reflect on their own policy and infrastructures in comparison to Britain and may have influenced developments after his departure. For example, South African pension officials told Gilbert that his lengthy residency provided them with a better understanding of British policy and administration.^152^ As the Ministry sent officials to visit and observe conditions overseas, South African and Australian officials similarly conducted visitations and reviews of overseas territories which are worthy of consideration as are the Ministry’s reciprocal treatment of foreign-born migrants residing in Britain. In addition, Australia, South Africa, and Britain were part of multinational veteran associations such as the FIDAC, the CIAMAC, and the BESL. Little research on their input into these transnational organizations, nor these multinational bodies’ impact on British, Australian, and South African veteran after-care, has been undertaken.

This article’s administrative approach mediated through the individual perspective of Gilbert hopes to encourage further research to better uncover the lived experience of Imperial Pensioners. There are undeniable challenges in studying these men. Migration historians attest to the shortage of primary materials dedicated to individual migrants and migrant communities in international archives.^153^ Yet, detailed and readily available sources exist to help reveal the individual experience of Imperial Pensioners in the 787 PIN 26 pension files of disabled Overseas First World War veterans held at the National Archives in London. These include a wealth of personal and experiential materials including cross-national correspondence, medical assessment reports and even interview transcripts.^154^ These pensioner records, used in tandem with bureaucratic records such as the reports mentioned above, will help improve our understanding of the Ministry of Pensions and disabled Great War veterans both at home and abroad.

Finally, further research is still needed into the ways in which “historically, people with disabilities have been powerless, marginalized socially, politically, economically and denigrated by dominant cultural values.”^155^ Yet, Imperial Pensioners were active, mobile and independent individuals willing to migrate thousands of miles in search for a better life. David Gerber cautions historians against repeating common public and literary representations of disabled people and populations through repeating narratives of “suffering and pain.”^156^ Gerber writes, “By shifting the perspective toward the experience of disability may we understand the nature of the veteran’s own agency in attempting to shape his relations to the state around his own conception of his needs and aspirations.”^157^ Disability history is a burgeoning field of research. Historians engaged in this field of study are now better able to contribute to this rebalancing by highlighting disabled people’s private and professional lives to better recognize their agency in improving their treatment and position within society.^158^ Further analysis of the hitherto overlooked Imperial Pensioner and other active and mobile disabled veteran communities will help to amplify how disability does not equate to inability.

## Footnotes

1 Memo written by L. S. Amery, 6 May 1925, PIN 15/1674 National Archives, Kew Gardens, London (hereafter NA); Letter from the Treasury to the Ministry of Pensions, 30 November 1920, T/126/4, NA.

2 Letter from Ministry of Pensions to the Treasury, 22 August 1924, PIN 15/1673, NA.

3 Ángel Alcade, “War Veterans as Transnational Actors: Politics, Alliances and Networks in the Interwar Period,” *European Review of History* 25 (2018): 505.

4 Gilbert mainly travelled by boat and rail to visit Australia, South Africa, India, France, Malta, the British West Indies, Nyasaland, Southern Rhodesia, Northern Rhodesia, Kenya, Zanzibar, Uganda, Tanganyika, New Zealand, Seychelles, Fiji, Egypt, Palestine, and Sudan.

5 Ana Carden-Coyne and Julie Anderson, “Enabling the Past: New Perspectives in the History of Disability,” *European Review of History* 14 (2007): 455.

6 This special virtual issue, entitled “Medical Care during the First World War,” was published online in 2014 and is available at https://academic.oup.com/jhmas/pages/virtual_issue.

7 This new methodology hopes to build upon the recent work of Mark Edele, Neil J. Diamant, and Martin Crotty who included Australian and British First World War veterans in their twentieth-century study of veteran state policy across multiple combatant nations. Martin Crotty, Neil J. Diamant, and Mark Edele, *The Politics of Veteran Benefits in the Twentieth Century* (Ithaca: Cornell University Press, 2020). See also Deborah Cohen, *The War Come Home: Disabled Veterans in Britain and Germany, 1914-1939* (Berkeley: University of California Press, 2001).

8 Alcade, “War Veterans as Transnational Actors,” 492-511; Julia Eichenberg and Natali Stegmann, “Divided by War, United by Welfare: The International Labour Organization Promoting War Invalids’ Internationalism,” *European Review of History* 29 (2022): 590-613; Julie M. Powell, *Bodies of Work: The First World War and the Transnational Making of Rehabilitation* (Cambridge: Cambridge University Press, 2022).

9 Julia Eichenberg and John Paul Newman, “Introduction,” in *The Great War and Veterans’ Internationalism*, ed. Julia Eichenberg and John Paul Newman (Basingstoke: Palgrave Macmillan, 2013), 3.

10 Canada hosted a unique Ministry branch office in Ottawa, established in 1926, to cater to around 11,500 Imperial Pensioners across North America. Military stations and barracks administered assessments and treatment in imperial territories such as India, Egypt, Sudan, and Palestine. British consulates became the primary intermediary for Imperial Pensioners in foreign countries without affiliation to the British Empire. Sixth Annual Report of the Ministry of Pensions, 1922-1923, 5.

11 Important distinctions among the three countries must be noted. These include, for example, geographic differences such as the vast contrast in size between Britain and Australia or cultural differences such as the British segregation of privates from officers in pension scales and even segregated treatment.

12 Ninth Annual Report of the Ministry of Pensions, 1925-1926, 24.

13 Peter Barham, *Forgotten Lunatics of the Great War* (New Haven: Yale University Press, 2004), 187; Cohen, *The War Come Home,* 57; Stephen R. Ward “Great Britain: Land Fit for Heroes Lost,” in *The War Generation,* ed. Stephen R. Ward (London: Kennikat Press, 1975), 3-37; Ian Beckett, Timothy Bowman, and Mark Connelly, *The British Army and the First World War* (Cambridge: Cambridge University Press, 2017), 163; Simple History YouTube Channel, “A Land Fit For Heroes? The Treatment of WW1 Veterans in Great Britain,” available at https://www.youtube.com/watch?v=iZ-OgzIRG9c&t=4s; Adam Powell, *Soldiering On: British Tommies After the First World War* (Cheltenham: The History Press, 2019).

14 Cohen, *The War Come Home,* 58-59.

15 Crotty, Diamant, and Edele, *Politics of Veteran Benefits in the Twentieth Century*, 93.

16 Poland’s rehabilitative and welfare systems emerged in the context of a nation re-emerging as a nation-state in the aftermath of the First World War. Its infrastructure was, therefore, subsequently hastily established. Anita Magowska, “The Unwanted Heroes: War Invalids in Poland After World War I,” *Journal of the History of Medicine and Allied Sciences* 69 (2016): 185-220.

17 Daniel Blackie and Alexia Moncrieff, “State of the Field: Disability History,” *Journal of the Historical Association* 107 (2022): 807-809.

18 David Swift and Oliver Wilkinson, “Introduction,” in *Veterans of the First World War: Ex-Servicemen and Ex-Servicewomen in Post-War Britain and Ireland,* ed. David Swift and Oliver Wilkinson (London: Routledge, 2019), 2.

19 Mayhew and Crenner, “Introduction to the Virtual Issue,” 4.

20 Martin Crotty and Mark Edele, “Total War and Entitlement: Towards a Global History of Veteran Privilege,” *Australian Journal of Politics & History* 59 (2013): 16.

21 Roy Hanes, “Introduction,” in *The Routledge History of Disability,* ed. Roy Hanes, Ivan Brown, and Nancy E. Hansen (London: Routledge, 2017), 4.

22 Crotty, Diamant, and Edele, *The Politics of Veteran Benefits in the Twentieth Century*, 7; Blackie and Moncrieff, “State of the Field,” 805.

23 Michelle Moyd, “African Military Historiography,” *War & Society* 42 (2023): 34.

24 George N. Njung, “Victims of Empire: WW1 Ex-Servicemen and the Colonial Economy of Wartime Sacrifices in Post-War British Nigeria,” *First World War Studies* 10 (2019): 51; Michelle Moyd, “Centring a Sideshow: Local Experiences of the First World War in Africa,” *First World War Studies* 7 (2016): 111-130; Julie Livingstone, “Insights From An African History of Disability,” *Radical History Review* 94 (2006): 111-216; Jos Raadschelders, *Handbook of Administrative History* (London: Routledge, 1998), 19.

25 Moyd, “African Military Historiography,” 36.

26 Njung, “Victims of Empire,” 49-67.

27 Dónal Hassett and Michelle Moyd, “Introduction: Writing the History of Colonial Veterans of the Great War,” *First World War Studies* 10 (2019): 1-11.

28 Elizabeth Bredberg, “Writing Disability History: Problems, Perspectives and Sources,” *Disability and Society* 14 (1999): 191.

29 Blackie and Moncrieff, “State of the Field,” 791.

30 Powell, *Bodies of Work,* 15.

31 Blackie and Moncrieff, “State of the Field,” 795.

32 There was a short period of chaotic administration of pension assessment and delay of payments in 1919 due to the mass demobilization of service personnel at the official cessation of the conflict. Employment responsibilities were also transferred to the Ministry of Labor to help reduce the Ministry of Pensions’ workload.

33 By comparison, there was broader dissatisfaction attached to the actual contents of pension legislation. The entire back catalogue of *The Legion* is available in the British Library.

34 Jessica Meyer, *Men of War: Masculinity and the First World War in Britain* (London: Palgrave Macmillan, 2009), 101.

35 Crotty, Diamant, and Edele, *Politics of Veteran Benefits in the Twentieth Century*, 105-106.

36 Kent Fedorowich, *Unfit for Heroes: Reconstruction and Soldier Settlement in the Empire Between the Wars* (Manchester: Manchester University Press, 1995).

37 Jean P. Smith, “From Promising Settler to Undesirable Immigrant: The Deportation of British-born in Interwar Australia and South Africa,” *Journal of Imperial and Commonwealth History* 46 (2018): 506.

38 Nineteenth Annual Report of the Ministry of Pensions, 1935-1936, Part Two, 5-6.

39 Pensions were paid in sterling or in local currency in line with current exchange rates. Kent Fedorowich and Andrew Thompson, “Introduction: Mapping the Contours of the British World: Empire, Migration and Identity,” in *Empire, Migration and Identity in the British World,* ed. Kent Fedorowich and Andrew Thompson (Manchester: Manchester University Press, 2013), 12; Nineteenth Annual Report of the Ministry of Pensions, 1935-1936, Part One, 1; Nineteenth Annual Report of the Ministry of Pensions, 1935-1936, Part Two, 5-6.

40 Gilbert’s report on Australia is also discussed in Michael Robinson, “No Man’s Land: Disability, Rehabilitation, Welfare Policy and the British Ex-Service Migrant in Australia, 1918–39,” *Social History of Medicine* 34 (2021): 222-225.

41 The Repatriation Commission replaced the preceding Department of Repatriation. For more information on the Department of Repatriation, see Kate Blackmore, *The Dark Pocket of Time: War, Medicine and the Australian State, 1914-35* (Adelaide: Lythrum Press, 2008), 59-76.

42 Report of the Repatriation Commission for the year ending 30 June 1926, 46.

43 Crotty, Diamant, and Edele, *Politics of Veteran Benefits in the Twentieth Century*, 15, 93; Crotty and Edele, “Total War and Entitlement,” 24.

44 Clem Lloyd and Jacqui Rees, *The Last Shilling: A History of Repatriation in Australia* (Melbourne: Melbourne University Press, 1994); Alistair Thomson, *Anzac Memories: Living with the Legend* (Oxford: Oxford University Press, 2013); Blackmore, *Dark Pocket of Time*, 108, 212-223, 231.

45 “Australia: Report,” PIN 15/1716, NA.

46 Ibid.

47 Carolyn Holbrook, *ANZAC: The Unauthorised Biography* (Sydney: New South Wales, 2014), 206.

48 Mark David Sheftall, *Altered Memories of the Great War: Divergent Narratives of Britain, Australia, New Zealand and Canada* (London: I. B. Tauris, 2010).

49 Thomson, *Anzac Memories*, 126.

50 “Australia: Report,” PIN 15/1716, NA.

51 Ibid.

52 Ibid.

53 Report of the Repatriation Commission for the year ending 30 June 1926, 8.

54 Report of the Repatriation Commission for the year ending 30 June 1923, 8.

55 There are three further important veteran issues unique to South Africa but which remain beyond the remit of this study. These issues include the simultaneous payment of disability pensions to Boer veterans who had previously fought against Britain, the payment of pensions to veterans of colonial units such as the “Cape Coloured Corps,” and the payment of South African veterans who enlisted in the British Armed Forces during the earlier stages of the First World War. Imperial Pensioners resident in South Africa: Mr Gilbert’s Report on his Visit to South Africa [hereafter “South Africa: Report”], PIN 15/2611, NA; “Pensions Administration in South Africa: Mr Milne’s Report,” PIN 15/1454, NA; Anri Delport, “Stumbling on Civvy Street: The Re-Adjustment of White South African Veterans to Life in Post-War Society, 1918-1928,” *Scientia Militaria: South African Journal of Military Studies* 44 (2016): 125.

56 “South Africa: Report,” PIN 15/2611, NA; Delport, “Stumbling on Civvy Street,” 125.

57 Ministry of Pensions Minute Sheet, 29 December 1927, PIN 15/2611, NA.

58 “South Africa: Report,” PIN 15/2611, NA.

59 Ibid.

60 “Disabled – Mutiles de Guerra – Afrique du sud Gouvernement,” MU 2/1/65, International Labor Organization Archive, Geneva, Switzerland.

61 “South Africa: Report,” PIN 15/2611, NA.

62 The requisite Ministry policy and procedure to be followed is provided in “War Pensions: Memorandum for Guidance of Ministry’s Overseas Pension Agents: South Africa,” PIN 14/67, NA.

63 “South Africa: Report,” PIN 15/2611, NA.

64 Like the British Legion, the British Empire Service League amalgamated existing veteran charity bodies into one consolidated organization. Unlike the British Legion, however, the British Empire Service League operated in an official capacity with the South African government paying for their property rental costs. In return, the charity took on the role akin to local war pensions committees in Britain acting as the first point of contact between the state and the veteran with initial inquiries or complaints and appeals. By Gilbert’s arrival, it had seventy branches representing around 7,000 veterans. Ministry of Pensions Minute Sheet, 29 December 1927; “South Africa: Report,” PIN 15/2611, NA; “Pensions Administration in South Africa: Mr Milne’s Report,” PIN 15/1454, NA.

65 “South Africa: Report,” PIN 15/2611, NA.

66 Ibid.

67 “Great War: Returned Soldiers: Payment of Pensions and Gratuities”; “Office of Military Pensions Office, 26 June 1926,” 1/WIL, South Africa National Library Special Collections, Cape Town, South Africa.

68 “South Africa: Report,” PIN 15/2611, NA.

69 Ibid.

70 “Pensions Administration in South Africa: Mr Milne’s Report,” PIN 15/1454, NA.

71 Ibid.; “South Africa: Report,” PIN 15/2611, NA.

72 Hilary Buxton, “Imperial Amnesia: Race, Trauma and Indian Troops in the First World War,” *Past & Present* 241 (2018): 221-258; Robinson, *Shell-Shocked British Army Veterans in Ireland,* 63, 202-204.

73 “Pensions Administration in South Africa: Mr Milne’s Report,” PIN 15/1454, NA; “South Africa: Report,” PIN 15/2611, NA.

74 Ministry of Pensions minute sheet, 31 January 1936, PIN 15/1458, NA; “South Africa: Report,” PIN 15/2611, NA.

75 Njung, “Victims of Empire,” 60.

76 Hassett and Moyd, “Introduction,” 1-11.

77 Fourth Annual Report of the Ministry of Pensions, 1920-1921, 8, 24, 35.

78 Its temporality also aimed to deter veterans from remaining in hospital after the benefits of continued medical/rehabilitative care had supposedly ceased.

79 Helen Bettinson, “Lost Souls in the House of Restoration: British Ex-Servicemen and War Disability Pensions, 1914–1930” (PhD diss., University of East Anglia, 2002), 208.

80 Tenth Annual Report of the Ministry of Pensions, 1926-1927, 2.

81 “Australia: Report,” PIN 15/1716, NA; “Australia: Report (Appendices),” PIN 15/671, NA.

82 “South Africa: Report,” PIN 15/2611, NA.

83 Western Australia reserved a separate exclusive ward for veterans in a public hospital. Also, Melbourne’s outpatient clinic was conducted at branch office of the Repatriation Commission rather than at its designated general hospital.

84 Report of the Repatriation Commission for the year ending 30 June 1922, 13-17; Report of the Repatriation Commission for the year ending 30 June 1926, 5.

85 “Repatriation Commission, Minute Paper,” September 1931, G193 File 3, State Archive, Sydney, Australia.

86 Calculated from “Australia: Report,” PIN 15/1716, NA.

87 “Australia: Report,” PIN 15/1716, NA.

88 Annual Report for Public Health Department, 1925, 42; Annual Report for Public Health Department, 1926, 63, South Africa National Library, Cape Town, South Africa.

89 “South Africa: Report,” PIN 15/2611, NA.

90 Ibid.

91 Ibid.

92 Ibid.

93 Ibid.

94 Ibid.

95 Ibid.

96 Barham, *Forgotten Lunatics of the Great War,* 278; Edgar Jones and Simon Wessely, “War Pensions (1900-1945): Changing Models of Psychological Understanding,” *British Journal of Psychiatry* 180 (2002): 376.

97 Barham, *Forgotten Lunatics of the Great War,* 273-278.

98 Ibid., 374; Fourth Annual Report of the Ministry of Pensions, 1920-1921, 9, 32-4; Edgar Jones and Simon Wessely, *Shell-Shock to PTSD: Military Psychiatry from 1900 to the Gulf War* (Hove: Psychology Press, 2005), 133; Director General to all regions, 28 September 1923, PIN 15/2946, NA; Provision of Treatment for Neurasthenia, 25 November 1921, PIN 15/56, NA.

99 Michael Robinson, “Definitely Wrong? The Ministry of Pensions’ Treatment of Mentally Ill Great War Veterans in Interwar British and Irish Society,” *War in History* 28 (2021): 86.

100 Barham, *Forgotten Lunatics of the Great War,* 104, 293, 327.

101 “Instruction Relating to the Classification and Treatment of Soldiers and Sailors as Service Patients,” PIN 15/896, NA.

102 Departmental Committee of Inquiry into the Machinery of Administration of the Ministry of Pensions Report to Ian Macpherson, MP, Minister of Pensions, 1921, 105; Two smaller hospitals for “insane” First World War veterans of “the hopeful type” were established in annexed buildings on asylum grounds operating along public facility lines. Long-term admissions remained commonplace and the two hospitals were closed in 1931 with remaining patients transferred back to civilian mental hospitals. Alice Brumby, “A Painful and Disagreeable Position: Rediscovering Patient Narratives and Evaluating the Difference Between Policy and Experience for Institutionalised Veterans with Mental Disabilities,” *First World War Studies* 6 (2015): 37-55.

103 Ninth Annual Report of the Ministry of Pensions, 1925-1926, 24.

104 Barham, *Forgotten Lunatics of the Great War*; Jennifer Farquharson, “Health and Hierarchy: Soldiers, Civilians and Mental Healthcare in Scotland, 1914–34,” *History of Psychiatry* 29 (2018): 79-95; Michael Robinson, *Shell-Shocked British Army Veterans in Ireland*, 185-226.

105 Richard Lindstrom, “The Australian Experience of Psychological Casualties in War, 1915-1939” (PhD diss., Victoria University of Technology, 1997), 184; Marina Larsson, “Families and Institutions for Shell-Shocked Soldiers in Australia after the First World War,” *Social History of Medicine* 22 (2009): 97-114; Report of the Repatriation Commission for the year ending 30 June 1922, 17.

106 Report of the Repatriation Commission for the year ending 30 June 1921, 16, 19.

107 Report of the Repatriation Commission for the year ending 30 June 1926, 15.

108 “Australia: Report,” PIN 15/1716, NA.

109 Larsson, “Families and Institutions for Shell-Shocked Soldiers in Australia after the First World War,” 101.

110 “Australia: Report,” PIN 15/1716, NA.

111 Delport, “Stumbling on Civvy Street,” 123.

112 “South Africa: Report,” PIN 15/2611, NA.

113 Commissioner for Returned Soldiers to all Returned Soldiers Advisory Boards, 26 February 1919, 1/AXA, Western Cape Archives and Records Service, Cape Town, South Africa.

114 “South Africa: Report,” PIN 15/2611, NA.

115 Ibid.

116 Ibid.

117 Ibid.

118 Ibid.

119 Ibid.

120 Ibid.

121 “Disabled Ex-Servicemen in Poor Law Institutions,” PIN 15/2530, NA.

122 Robinson, *Shell-Shocked British Army Veterans in Ireland,* 194, 198-199.

123 “South Africa: Report,” PIN 15/2611, NA.

124 “Mr Gilbert’s Report on his Visit to New Zealand,” PIN 15/1798, NA.

125 “Australia: Report,” PIN 15/1716, NA.

126 For example, a prolonged and patient period of psychotherapy with one neurasthenic veteran enabled an unlocking of repressed traumatic memories and a subsequent drastic improvement in the veteran’s severe psychoneurotic symptoms. Report of the Repatriation Commission for the year ending 30 June 1925, 16-21.

127 Robinson, “Definitely Wrong,” 76-80.

128 Robinson, *Shell-Shocked British Army Veterans in Ireland,* 110.

129 Hanes, “Introduction,” 4.

130 British Legion Journal, 1923, 3, 43; Joanna Bourke, “The Battle of the Limbs: Amputation, Artificial Limbs and the Great War in Australia,” *Australian Historical Studies* 29 (1998): 49–67. For more information on the design of the British metallic limb, see Mary Guyatt, “Better Legs: Artificial Limbs for British Veterans of the First World War,” *Journal of Design History* 14 (2001): 307–325; T. J. Mitchell and G.M. Smith, *Medical Services, Casualties and Medical Statistics of the Great War* (London: HM Stationary Office, 1931), 339.

131 Nineteenth Annual Report of the Ministry of Pensions, 1935-1936, Part Two, 20.

132 Seth Koven, “Remembering and Dismemberment: Crippled Children, Wounded Soldiers and the Great War in Great Britain,” *American Historical Review* 99 (1994): 1195.

133 Roehampton Hospital continued in this role, even acting as an agent on behalf of the Ministry of Health, assisting veterans and civilians after World War II. Twenty-First Annual Report of the Ministry of Pensions, 1937-1938, 14; Twenty-Third Annual Report for the Ministry of Pensions, 1939-1948, 22.

134 “Australia: Report,” PIN 15/1716, NA.

135 Bourke, “Battle of the Limbs,” 49-67.

136 Report of the Repatriation Commission for the year ending 30 June 1925, 30-32; Report of the Repatriation Commission for the year ending 30 June 1926, 13.

137 Ibid., 56-57.

138 Margaret Hutchison, “Old Age is Not a War Disability: Debating Aged Care for Nurses of World War I in Post-1945 Australia,” *Australian Historical Studies* 52 (2021): 63-78.

139 “South Africa: Report,” PIN 15/2611, NA.

140 Ibid.

141 Ibid.

142 Ibid.

143 Ibid.

144 Annual Report for Public Health Department, 1926, 63, South Africa National Library, Cape Town, South Africa, 64.

145 “South Africa: Report,” PIN 15/2611, NA.

146 “Imperial Pensioners in South Africa: Mr. W.A. Christie’s visit September 1935,” PIN 15/1457, NA.

147 Crotty, Diamant, and Edele, *Politics of Veteran Benefits in the Twentieth Century*, 11.

148 Ibid., 93.

149 Grace Huxford, Ángel Alcalde, Gary Baines, Olivier Burtin, and Mark Edele, “Writing Veterans’ History: A Conversation on the Twentieth Century,” *War & Society* 38 (2019): 122-123; Swift and Wilkinson, “Introduction,” 1.

150 Kirsti Bohata, Alexandra Jones, Mike Mantin, and Steven Thompson, *Disability in Industrial Britain: A Cultural and Literary History of Impairment in the Coal Industry, 1880-1948* (Manchester: Manchester University Press, 2019), 106.

151 Christopher Pierson and Matthieu Leimgruber, “Intellectual Roots,” in *The Oxford Handbook of Disability History,* ed. Christopher Pierson and Matthieu Leimgruber (Oxford: Oxford University Press, 2017), 40-41.

152 The Secretary of Finance in charge of administering veteran pensions in South Africa agreed with Gilbert’s suggestion that the South African medical system would benefit from replicating British policy by deducting money from an inpatient’s disability pension. The Secretary of Finance indicated his desire to explore the policy further to help prevent the aforementioned costly bed-blocking. “South Africa: Report,” PIN 15/2611, NA.

153 Eric Richards, *Destination Australia: Migration to Australian Since 1901* (Sydney: University of New South Wales Press, 2008), 10; Michael Roe, *Australia, Britain, and Migration, 1915-1940: A Study of Desperate Hopes* (Cambridge: Cambridge University Press, 1995), 254; Tony Kushner, “Alienated Memories: Migrants and the Silences of the Archive,” in *Memory and History: Understanding Memory as Source and Subject,* ed. Joan Tumblety (London: Routledge 2013), 177.

154 There are 22,823 veteran files the majority of which incorporate UK-based veterans. Jessica Meyer, “He Does Not Appear To Have Done Much Useful Work Since He Was Wounded: Age, Disability and the History of Masculinity,” *Tijdschrift voor genderstudies* 25 (2022): 43.

155 Jeanne Hayes, and Elizabeth M. Hannold, “The Road to Empowerment: A Historical Perspective on the Medicalization of Disability,” *Journal of Health and Human Services Administration* 30 (2007): 356.

156 David Gerber, “Preface: The Continuing Relevance of the Study of Disabled Veterans,” in *Disabled Veterans in History,* ed. David Gerber (Ann Arbor: University of Michigan Press, 2012), xiv.

157 David Gerber, “Disabled Veterans, the State, and the Experience of Disability in Western Societies, 1914-1950,” *Journal of Social History* 36 (2003): 901.

158 David M. Turner and Daniel Blackie, *Disability in the Industrial Revolution: Physical Impairment in British Coalmining, 1780–1880* (Manchester: Manchester University Press, 2018), 200-202; Bohata, Jones, Mantin, and Thompson, *Disability in Industrial Britain,* 77.

